# DPSP: a multimodal deep learning framework for polypharmacy side effects prediction

**DOI:** 10.1093/bioadv/vbad110

**Published:** 2023-08-16

**Authors:** Raziyeh Masumshah, Changiz Eslahchi

**Affiliations:** Department of Computer and Data Sciences, Faculty of Mathematical Sciences, Shahid Beheshti University, Tehran 1983969411, Iran; Department of Computer and Data Sciences, Faculty of Mathematical Sciences, Shahid Beheshti University, Tehran 1983969411, Iran; School of Biological Sciences, Institute for Research in Fundamental Sciences (IPM), Tehran 193955746, Iran

## Abstract

**Motivation:**

Because unanticipated drug–drug interactions (DDIs) can result in severe bodily harm, identifying the adverse effects of polypharmacy is one of the most important tasks in human health. Over the past few decades, computational methods for predicting the adverse effects of polypharmacy have been developed.

**Results:**

This article presents DPSP, a framework for predicting polypharmacy side effects based on the construction of novel drug features and the application of a deep neural network to predict DDIs. In the first step, a variety of drug information is evaluated, and a feature extraction method and the Jaccard similarity are used to determine similarities between two drugs. By combining these similarities, a novel feature vector is generated for each drug. In the second step, the method predicts DDIs for specific DDI events using a multimodal framework and drug feature vectors. On three benchmark datasets, the performance of DPSP is measured by comparing its results to those of several well-known methods, such as GNN–DDI, MSTE, MDF–SA–DDI, NNPS, DDIMDL, DNN, DeepDDI, KNN, LR, and RF. DPSP outperforms these classification methods based on a variety of classification metrics. The results indicate that the use of diverse drug information is effective and efficient for identifying DDI adverse effects.

**Availability and implementation:**

The source code and datasets are available at https://github.com/raziyehmasumshah/DPSP.

## 1 Introduction

Polypharmacy (the concurrent use of multiple drugs) has become a successful strategy for combating complex or co-existing diseases (Masnoon *et al.* 2017, Guillot *et al.* 2020, Masumshah *et al.* 2021, Tanvir *et al.* 2022, Yao *et al.* 2022, Lin *et al.* 2022a). However, in some cases, drug combinations can cause Adverse Drug Reactions (i.e. side effects) (Shah and Hajjar 2012, Zitnik *et al.* 2018, Nováček and Mohamed 2020, Bumgardner *et al.* 2021, Lin *et al.* 2022b). Drug–drug interactions (DDIs) may alter the activity of the drugs with increasing or decreasing the effect of drugs, and in the worst case may lead to death (Masumshah *et al.* 2021, Tanvir *et al.* 2022).Therefore, the effective detection of adverse DDIs is an urgent problem in human health (Masoudi-Nejad *et al.* 2013, Liu *et al.* 2017, Yan *et al.* 2018, Lin *et al.* 2020, Kim and Shin 2023, Liu *et al.* 2023, Wang *et al.* 2023). A general source for known DDIs is through the experiments (Tari *et al.* 2010, Kim and Nam 2022). However, identifying polypharmacy side effects *in vitro* and *in vivo* is impractical in terms of cost and time (Rohani and Eslahchi 2019, Deng *et al.* 2020, Feng *et al.* 2020, Al-Rabeah and Lakizadeh 2022, Kim and Nam 2022). Thus, many computational models are developed to detect DDIs. The existing methods are roughly divided into two types: classification-based methods and similarity-based methods (Yan *et al.* 2018, Zitnik *et al.* 2018, Han *et al.* 2021). Classification-based methods consider DDI prediction as binary classification task (Cheng and Zhao 2014, Yan *et al.* 2018, Han *et al.* 2021, Yao *et al.* 2022). In these techniques, the presence or absence of interactions are considered as positive and negative samples, respectively, and used to train classification models like KNearest-Neighbor (KNN), Logistic Regression (LR), and Random Forest (RF) (Breiman 2001, Mitchell 2005, Peterson 2009, Cheng and Zhao 2014, Denisko and Hoffman 2018). In the recent classification-based method, Yao *et al.* (2022) proposed a new knowledge graph embedding method, denoted by MSTE, which converts the side effects prediction task into the link prediction problem. In contrast, similarity-based methods assume that similar drugs may interact with the same drug (Mitchell 2005, Peterson 2009, Zhang *et al.* 2017, Zitnik *et al.* 2018). The GNN–DDI method generated a drug embedding vector and used a graph neural network to predict polypharmacy side effects prediction (Al-Rabeah and Lakizadeh 2022). Lin proposed the MDF–SA–DDI method based on multi-source drug fusion, multi-source feature fusion, and transformer self-attention mechanism to perform latent feature fusion (Lin *et al.* 2022b). Masumshah *et al.* (2021) presented the NNPS method based on a dimension reduction technique and used new features to predict DDIs. The DDIMDL method proposed by Deng *et al.* (2020) constructs sub-models based on diverse drug features and by adopting a deep neural network (DNN) framework, combines the sub-models and feeds to fully connected neural networks. Hou *et al.* (2019) built a DNN model and predicted DDI events by using SMILES features. Ryu *et al.* (2018) presented a deep learning approach, named DeepDDI, based on chemical substructures to predict crucial DDIs. While previous methods have been effective in predicting polypharmacy side effects, certain approaches, such as GNN–DDI, DDIMDL, and MSTE suffer from imbalanced distribution among different events, which can negatively impact prediction accuracy. Also, NNPS only uses a small number of features and may not capture the full complexity of DDI, and the quantity and the caliber of training data in the DeepDDI method might be a restriction on its performance. Furthermore, recently methods like MDF–SA–DDI, DDIMDL, and MSTE, have very complicated architectures and need a lot of computing power. Given the limitations and drawbacks of existing methods for predicting drug interactions, it is necessary to develop a new method that can address these limitations and provide a more comprehensive and reliable approach for predicting drug interactions. Using five different drug features, including mono side effects, target proteins, enzymes, chemical structure, and pathways, and feeding them into a neural network architecture optimized for DDI prediction, our proposed method addresses some of these limitations. Other methods are different from ours in a number of ways, but the predictive neural network architecture is the most important difference. Our method uses a neural network architecture with only three hidden layers that are fairly simple. This simple architecture reduces overfitting and improves interpretability while maintaining high prediction accuracy. In addition, our neural network is able to rapidly learn due to the use of five distinct drug characteristics to identify similarities between drugs, which are then fed into the network. This speed of learning and prediction is especially remarkable when compared to the longer training and inference times required by more complex architectures employed by earlier methods. In this study, we develop a Deep learning framework for Polypharmacy Side effects Prediction (DPSP) based on multiple features. DPSP achieves better results in comparison with 10 well-known methods in terms of accuracy, complexity, and running time speed. The required datasets and the details of the DPSP method are described in the next section. In Section 3, six criteria are used to compare the results of DPSP with those of other methods. Finally, the conclusion and future works are provided in Section 4.

## 2 Materials and methodology

**Figure 1.**
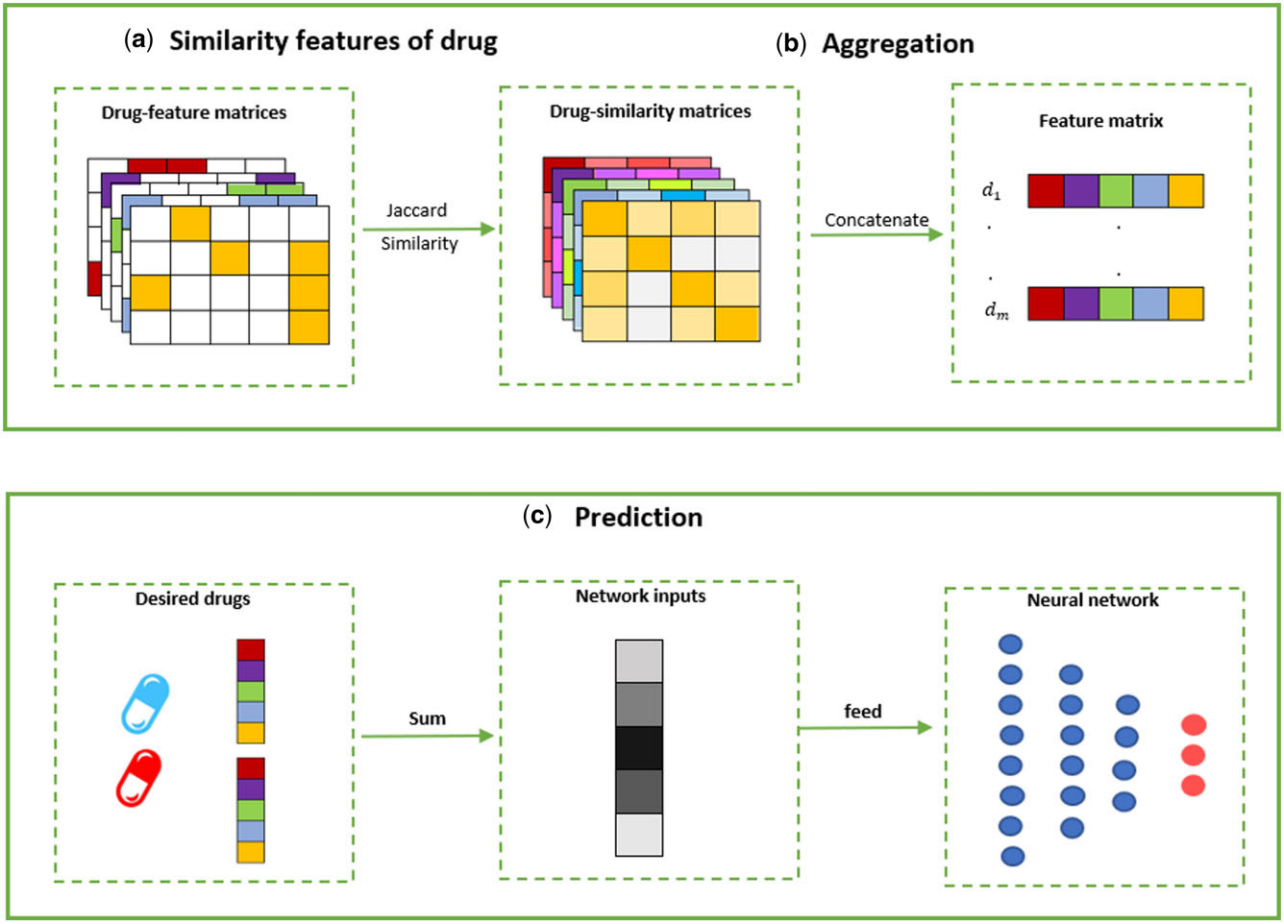
The scheme of DPSP workflow. (a) Constructing similarity feature matrix by using multiple information of drugs. (b) Concatenating similarity matrices to obtain the feature matrix. (c) Sum of the *i*-th and *j*-th rows of the drug feature matrix and feed to the neural network to predict the interaction between drug $d_{i}$ and $d_{j}$.

In this section, we formulate the problem and present the details of the method, including the input format and all of its steps. The overall framework of DPSP is illustrated in Fig. 1.

### 2.1 Problem formulation

Given a set of drugs $D=\left\{d_{1},\ldots ,d_{m}\right\}$ and a set of event types $E=\left\{e_{1},\ldots ,e_{n}\right\}$, where *m* and *n* are the total number of drugs and events, respectively. A DDI prediction task can be regarded as a probability function f:D×D→E, which predicts the probability of each type of event that will occur between the two drugs.

### 2.2 Datasets description

DrugBank is a comprehensive database that provides information about 12 151 drugs, including experimental and FDA-approved drugs (Knox *et al.* 2011, Deng *et al.* 2020, Al-Rabeah and Lakizadeh 2022, Lakizadeh and Babaei 2022). KEGG is an integrated database for biological interpretation (Knox *et al.* 2011, Kanehisa and Goto 2000, Kanehisa *et al.* 2017, Ryu *et al.* 2018, Hou *et al.* 2019). Pubchem consists of diverse types of substructures with values of zero or one, which denotes the absence or presence of a certain substructure (Kim *et al.* 2016). The OFFSIDES and Side Effect Resource (SIDER) databases were integrated to derive the side effects of individual drugs (mono side effects) (Tatonetti *et al.* 2012, Kuhn *et al.* 2016). TWOSIDES provides a reliable and comprehensive database for DDIs (Tatonetti *et al.* 2012). In this study, to verify the robustness of DPSP, we validate it on different benchmark datasets. We used the benchmark datasets that used in previous studies (Deng *et al.* 2020, Al-Rabeah and Lakizadeh 2022, Lakizadeh and Babaei 2022, Lin *et al.* 2022c). The first benchmark (DS1) contains 572 drugs with 37 264 DDIs, which are associated with 65 types of events. This benchmark includes five types of features which the four drug features, including targets, enzymes, chemical substructures, and pathways are selected from the DrugBank, KEGG, and Pubchem databases, and the mono side effects are collected from the OFFSIDES and SIDER databases. The second benchmark (DS2) contains 1258 drugs with 161 770 DDIs, which are associated with 100 types of events. This benchmark includes three types of features include targets, enzymes, and chemical substructures, which are collected from the DrugBank. The third dataset contains 645 drugs and 63 473 drug interactions, wherein each pair of drugs may have multiple adverse effects. For drug combinations with multiple adverse effects, we only considered the side effect with the highest frequency among the others. Therefore, there are 185 adverse effects. This benchmark consists of two kinds of characteristics: mono side effects and targets collected from TWOSIDES. The representation of DDI events in two benchmark datasets DS1 and DS2, are defined as a four-tuple structure: (drug A, drug B, mechanism, action) which the “mechanism” in this tuple refers to the effects of drugs in terms of the metabolism, the severity of adverse effects, the serum concentration, the therapeutic efficacy, etc., and reducing or increasing the effects are represented in the “action” term. DS3 are depicted as a triple (polypharmacy adverse effect, drug A, and drug B). Further details about these datasets are available in Table 1.

**Table 1.** Details of benchmarks.

Benchmark name	Benchmark details	Benchmark features	Reference
DS1	No. drugs = 572 No. interactions = 37 264 No. events = 65	No. side effects = 9991 No. targets = 1162 No. enzymes = 202 No. chemical substructure = 881 No. pathways = 957	Deng *et al*. 2020, Al-Rabeah and Lakizadeh 2022, Lakizadeh and Babaei 2022
DS2	No. drugs = 1258 No. interactions = 161 770 No. events = 100	No. targets = 1651 No. enzymes = 316 No. chemical substructure = 2040	Lin *et al*. 2022c
DS3	No. drugs = 645 No. interactions = 63 473 No. events = 185	No. side effects = 10 184 No. targets = 8934	Masumshah *et al*. 2021

### 2.3 The DPSP method

The architecture of DPSP is depicted in Fig. 1, which is composed of two main steps: constructing drug features and DDI prediction.

#### 2.3.1 Constructing drug features

In general, the feature matrix construction section consists of feature extraction module, and aggregation module.

- **Feature extraction module**: As shown in Fig. 1a, DPSP considers a set of features $F=\{f_{1},\ldots ,f_{p}\}$ and a feature matrix was constructed based on each feature, where *p* is the total number of features. In DS1, there are five feature matrices of the drugs, including the mono side effects, targets, enzymes, chemical substructures, and pathways. In another dataset (DS2), there are three feature matrices of the drugs, containing the targets, enzymes, and chemical substructures. In DS3, there are mono side effects and targets feature. In each matrix, each row represents a drug and each column represents a feature. Two types of entries, zero or one, indicate the absence or presence of the corresponding feature for each drug. For example, in the case of the mono side effects matrix in DS1, we have 9991 mono side effects, and so 9991 columns corresponding to these side effects. The entry correspond to the row $d_{i}$ and column $c_{j}$ has a value of one if drug $d_{i}$ has side effect $c_{j}$ and zero otherwise. After obtaining the feature matrices, the similarity matrices for each feature based on Jaccard similarity are constructed (Fig. 1a). The Jaccard similarity is defined as below:
  (1) $$J(X_{d_{i}},X_{d_{j}})=\frac{\left|X_{d_{i}}\cap X_{d_{j}}\right|}{\left|X_{d_{i}}\cup X_{d_{j}}\right|}=\frac{\left|X_{d_{i}}\cap X_{d_{j}}\right|}{\left|X_{d_{i}}|+|X_{d_{j}}|-|X_{d_{i}}\cap X_{d_{j}}\right|},$$

$X_{d_{i}}$ presents, for a certain feature matrix, the set of columns whose entries in the row $d_{i}$ of that feature matrix have the value one. In Equation (1), $\left|X_{d_{i}}\cap X_{d_{j}}\right|$ denote the number of common elements of $X_{d_{i}}$ and $X_{d_{j}}$, and $\left|X_{d_{i}}\cup X_{d_{j}}\right|$ denote the number of union elements of $X_{d_{i}}$ and $X_{d_{j}}$, respectively. Therefore, for each pair of drugs in each feature, the similarity between the two drugs will be calculated based on the above equation. So, we will have five similarity matrices of size 572×572, three matrices of size 1258×1258, and two matrices of size 645×645 in DS1, DS2, and DS3, respectively.
- **Aggregation module**: At this step, by concatenating the similarity matrices, the main drug feature matrix has been calculated (Fig. 1b). Therefore, the drug feature matrix in DS1 will be of dimension 572×2860, and in DS2 will be of dimension 1258×3774, and in DS3 will be of dimension 645×1290. The rows of the resulting matrix represent the IDs of the drugs, while the columns contain information about the features.

#### 2.3.2 DDI prediction

In Section 2.3.1, a unique feature vector for each drug was constructed. The purpose of this section is to express the architecture of the model, which is a DNN, and finally, predict the DDIs events. A DNN is an Artificial Neural Network with an input layer, multiple hidden layers, and an output layer (Nair and Hinton 2010, Schmidhuber 2015, Emmert-Streib *et al.* 2020). Parameters can affect the performance of our DPSP model. Therefore, we investigate the number of hidden layers, the number of neurons, the activation function, and the dropout rate based on the following steps to achieve the best performance.

- The number of hidden layers: {1,2,3}
- The number of neurons in hidden layers: {128,256,512}
- Activation functions: {Rectified Linear Unit (ReLU), Sigmoid, and Softmax}
- The dropout rate: {0.1,0.2,0.3,0.4,0.5}.

We trained several networks based on these parameters and represented them in Supplementary Tables S1–S6. As shown in these tables, three hidden layers with 512, 256, and 128 neurons, respectively, with a dropout rate of 0.3, utilizing the ReLU (Nair and Hinton 2010, Srivastava *et al.* 2014, Nwankpa *et al.* 2018) as the activation function for the first hidden layer, and Sigmoid activation function for other hidden layers, and the Softmax for output layer has the best results (Narayan 1997, Kouretas and Paliouras 2019). Now for a given drug pair $\left(d_{i},d_{j}\right)$, *i*-th and *j*-th rows of the drug feature matrix are summed and fed to the considered fully connected neural network (Fig. 1c). Then, we adopt binary-cross-entropy (Zhang and Sabuncu 2018) as the loss function and the training iteration (epoch) set to 100 with a batch size of 128. Adam optimizer (Kingma and Ba 2014) was used as the optimization algorithm to train the network. Finally, the network returns the probability of occurrence of each DDIs event. The pseudocode of DPSP is shown in Algorithm 1.

Algorithm 1 The pseudocode of DPSP algorithm

**Requite:** All features of drugs $F=\{f_{1},\ldots ,f_{p}\}$ including mono side effects, targets, enzymes, chemical substructure, and pathways;

**Ensure:** Prediction results.

1: **for** all i∈m**do**

2:  Calculate Jaccard similarity of drug features to measure drug similarity;

3:  Concatenate drug similarities;

4: Put the sum of *i*-th and *j*-th feature vectors as the input of the DNN model;

5: The model outputs the probability of occurrence of each DDI’s event;

6: The event with maximum probability will be selected;

## 3 Experiments and results

There are two main tasks in DDI prediction, first is identifying the absence or presence the interaction between the drugs. The second is determining the type of interaction among the drugs. In this article, we deal with the second case, and split the drug pairs into training and test sets, and employ 5-fold cross-validation to evaluate the DDI prediction task. In each fold, one subset is considered as the test set and the rest as training. For validating the performance of DPSP, we investigated it on two datasets (DS1, DS2, and DS3). We compared our method with 10 well-known methods GNN–DDI, MSTE, MDF–SA–DDI, NNPS, DDIMDL, DNN, DeepDDI, KNN, LR, and RF. We adopt the 5-fold cross-validation for 100 iterations and use the average of the results to assess the performance of all methods. To ensure a fair comparison and obtain optimal performance for the compared methods, we employed a 5-fold cross-validation method and conducted hyperparameter tuning for each of the compared methods using the 5-fold cross-validation. We chose the hyperparameters that yielded the greatest performance for each method, and the outcomes of the average of accuracy (ACC), area under the precision–recall curve (AUPR), area under the receiver-operating characteristic curve (AUROC), *F*_score, precision, and recall values, and the number of false negatives (FNs) along with the number of false positives (FPs) of all methods on DS1, DS2, and DS3 are shown in Table 2 and Supplementary Tables S7 and S8, respectively. According to Table 2 and Supplementary Tables S7 and S8, DPSP achieves better results than other well-known methods in terms of all criteria. DPSP outperforms both the similarity-based methods and the classification-based methods. Improvement in performance in each metric score of our method DPSP with respect to the highest score of the baseline methods are equal to 1.64% in ACC, 0.64% in AUPR, 0.05% in AUROC, 3.56% in *F*_score, 1.03% in precision, 3.52% in recall in DS1, and 0.16% in ACC, 0.14% in AUPR, 0.02% in AUROC, 0.04% in *F*_score, 0.16% in precision, and 0.43% in recall in DS2, and 1.11% in ACC, 2.72% in AUPR, 0.25% in AUROC, 0.57% in *F*_score, 0.75% in precision, and 0.24% in recall in DS3. The difference between DPSP performance with other methods in terms of *F*_score, precision, and recall is outstanding. To compare the results of DPSP more precisely, we compare it to the results of the best method between all methods (GNN–DDI) with more details. Supplementary Figs S1–S3 illustrate the boxplots of the *F*_score, precision, and recall criteria in all events by DPSP and GNN–DDI methods on DS1, DS2, and DS3 in 100 iterations, respectively. As shown in Supplementary Figs S1–S3, the range of variation of the *F*_score, precision, and recall criteria in both datasets for DPSP method are less than the range of variation of the *F*_score, precision, and recall criteria for the GNN–DDI method, which is the evidence of good performance and robustness of DPSP.

**Table 2.** Results of comparison of the DPSP with some of the machine-learning methods on DS1.^a^

Method	ACC	AUPR	AUROC	*F*_score	Precision	Recall	FP	FN
DPSP	**0.9344**	**0.9773**	**0.9990**	**0.9309**	**0.9309**	**0.9309**	**2573**	**2573**
GNN–DDI	0.9180	0.9709	0.9985	0.8999	0.8999	0.8999	3691	3691
MSTE	0.8584	0.9343	0.9981	0.8470	0.8470	0.8470	5698	5698
MDF–SA–DDI	0.9121	0.9657	0.9989	0.8832	0.8832	0.8832	4349	4349
NNPS	0.9113	0.9699	0.9989	0.8815	0.8815	0.8815	4413	4413
DDIMDL	0.8757	0.9353	0.9977	0.8277	0.8277	0.8277	6417	6417
DNN	0.8797	0.9134	0.9963	0.8046	0.8046	0.8046	7278	7278
DeepDDI	0.8371	0.8899	0.9961	0.7274	0.7274	0.7274	10 115	10 115
KNN	0.7201	0.7854	0.9821	0.7333	0.7333	0.7333	9935	9935
LR	0.7315	0.7988	0.9939	0.5340	0.5340	0.5340	17 362	17 362
RF	0.7756	0.8515	0.9955	0.7214	0.7214	0.7214	10 379	10 379

a Bold numbers show the best performance for each criterion.

Additionally, to disregard avoiding the overfitting arising from hyperparameter tuning, we used nested cross-validation with an outer-loop of 5-fold and an inner-loop of 5-fold to further tune the hyperparameters for our method. The results of the nested cross-validation for our method are presented in Supplementary Table S9. We observed that the results were very similar in both the nested cross-validation and 5-fold cross-validation methods, indicating that our method is robust to dataset splits in all DS1, DS2, and DS3.

### 3.1 Feature evaluation

In this section, we do two different evaluations based on the feature sets. In the first step of, we evaluated the significance of the features utilized by our DDI prediction model. To accomplish this, we computed the true positives (TP) and FPs for each combination of features to determine the effect of removing each feature. In particular, we computed the TP and FP results for the procedure utilizing all features and compared them to the results obtained after excluding each feature. After removing one of the features, including mono side effect, target, enzyme, chemical substructure, and pathway, the results of the DPSP method for DS1 are presented in Table 3, Supplementary Table S10, and the Venn diagrams in Supplementary Figs S4–S13. All of these features were discovered to be essential for attaining high model performance, as demonstrated by the findings. When each feature was removed and the DPSP method was re-executed with the four remaining features, model performance decreased significantly across all evaluation criteria. Consequently, this result demonstrated that the side, target, enzyme, pathway, and smile features all contributed to the overall performance of our method, with the pathway feature having the greatest impact on the outcomes. We have conducted comparable investigations with DS2. Once more, it is evident that all three characteristics of this dataset, targets, enzymes, and chemical substructures are necessary for accomplishing the goal of outstanding model performance. When each feature was removed and the DPSP method was re-executed with the remaining two features, model performance decreased significantly across all evaluation criteria. With chemical substructure features having the greatest impact on results (see Supplementary Tables S11 and S12 and Venn diagrams in Supplementary Figs S14–S19). In DS3, it is necessary to have both mono side effects and targets in order to achieve a high level of model performance. When each feature was removed and the DPSP method was re-executed with the remaining feature, model performance across all evaluation criteria decreased significantly. With the mono side effect feature having the greatest influence on the outcomes (see Supplementary Tables S13 and S14 and the Venn diagrams in Supplementary Figs S20–S23). Consequently, it would be instructive to evaluate the performance of the method utilizing only the most significant feature type. Therefore, our procedure was implemented using only the pathway feature in DS1, the chemical substructure feature in DS2, and the mono side effect feature in DS3. The results show that pathway information, chemical substructure, and mono side effect alone were insufficient to attain high performance (see Supplementary Tables S15–S17). This indicates that multiple categories of features must be combined to achieve optimal performance. The second criterion for evaluation is based on the size of the features. Due to the large size of the feature matrix, we are employing dimension reduction techniques to reduce its size. To achieve this, we use principal components analysis (PCA), AutoEncoder (AE), and Entropy to reduce the size of the input feature matrix of the DPSP algorithm. In this section, we looked at two distinct scenarios: (i) applying the three dimension reduction method to each of the similarity matrices and then concatenating them; and (ii) concatenating the similarity matrices first and then applying the three dimension reduction method to the concatenated matrix (which we used directly in the method). In both instances, the minimum number of principal components necessary to account for 95% of the variance is selected. For the other two dimension reduction techniques, we deemed the quantity of features to be comparable to what PCA yielded. In the first scenario, and for the DS1 dataset, the size of each Jaccard similarity matrix for mono side effects, targets, enzymes, chemical substructures, and pathways is reduced to 572×481, 572×186, 572×12, 572×11, and 572×173, respectively. In the second scenario, the feature matrix with dimension 572×2860 is reduced to 572×107. On DS2 and DS3, identical scenarios are utilized. The results of these cases are presented in Table 4 and Supplementary Tables S18–S25, which demonstrate that all of these dimensionality reduction techniques resulted in a performance decrease when applied to our method for all datasets. Nevertheless, our experiment revealed that the efficacy of our method decreased significantly when we employed the AE and Entropy dimensionality reduction methods as opposed to PCA. To demonstrate that dimensionality reduction can result in information loss, we applied the PCA method to all of the methods with which our results were compared, and the results are presented in Supplementary Tables S26–S31. In these tables, we can see that the PCA method resulted in a decrease in efficacy for each of the other methods. Our findings indicate that the original feature space is already highly informative for all methods and that reducing the dimensionality may result in a loss of information and a performance decrease. Data availability is an essential aspect of machine-learning and deep learning techniques. Therefore, we gathered information on the availability of data, including the number of zero and non-zero elements of each feature in each dataset, as shown in Supplementary Table S32 through S34. As seen in these tables, there are numerous zero elements. Therefore, for each feature, we determined the amount of similarity between each pair of drugs, and then incorporated this information into our model as a new feature. An essential aspect of our research is determining the impact of various feature combinations on our method’s performance. In order to provide a finer-grained analysis of the impact of various feature combinations, we reported results based on all 5!, 3!, and 2! feature combinations in DS1, DS2, and DS3, respectively. In Supplementary Tables S35–S38, the best-performing feature combinations for DS1, DS2, and DS3 are (M + T + E + P + S), (T + E + P + S), and (M + T), so we considered these combinations for our model in each dataset.

**Table 3.** This table demonstrates the significance of DS1 dataset features by removing one feature and evaluating the results of the DPSP method using the remaining features based on all evaluation criteria.

Excluded feature	ACC	AUPR	AUROC	*F*_score	Precision	Recall
Mono side effect	0.9283	0.9744	0.9989	0.8957	0.8957	0.8957
Target	0.9319	0.9752	0.9985	0.8778	0.8778	0.8778
Enzyme	0.9244	0.9727	0.9978	0.88800	0.8800	0.8800
Chemical substructure	0.9200	0.9767	0.9982	0.8742	0.8742	0.8742
Pathway	0.8070	0.8906	0.9969	0.7582	0.7582	0.7582

**Table 4.** The results of performing PCA dimensionality reduction technique on DS1.

PCA	ACC	AUPR	AUROC	*F*_score	Precision	Recall
Scenario 1	0.9264	0.9734	0.9989	0.8919	0.8919	0.8919
Scenario 2	0.8942	0.9573	0.9987	0.8279	0.8279	0.8279

## 4 Discussion and conclusion

Due to the side effects caused by the simultaneous use of medicinal compounds, it is impossible to screen all pairs of drugs in terms of time and cost. So, computational methods are developed to predict DDI events. In this article, we obtain the DDI data from the DrugBank, KEGG, PubChem, SIDER, OFFSIDES, and TWOSIDES and consider three types of datasets (DS1, DS2, and DS3), which classifies DDI-associated events into 65, 100, and 185 events, respectively. To elucidate, the events within our datasets serve as class labels. We solve a 65-class problem in DS1, 100-class problem in DS2, and 185-class problem in DS3. As previously mentioned, other methods utilize pairings of drugs available in each event to train and validate their models. We divided the data according to standard procedures, allocating 80% for model training and 20% for model assessment for each event. The results of all considered methods indicate that the models have been adequately trained, and our comparison indicates that our model trains better than the other method for these multiclass problems. For the prediction of polypharmacy side effects, we utilize DPSP, a DNN framework that employs multiple drug features. On DS1, DS2, and DS3 datasets, the model achieves excellent performance across all criteria. Supplementary Table S39 displays the execution time of each method on the DS1, DS2, and DS3 datasets. This table demonstrates that the DPSP method has the shortest execution time, 20 min on DS1 and 2 h on DS2, and 25 min on DS3. These execution times demonstrate the superior performance of this method compared to other methods. By utilizing an efficient algorithm and optimized hyperparameters, our method seeks to strike a balance between speed and accuracy. While we recognize that there may be tradeoffs between accuracy and speed in any machine-learning method, we have conducted extensive experiments to demonstrate that our approach is effective at attaining both high accuracy and computational efficiency. In our experiments, our method obtained accuracy comparable to or exceeding that of existing methods, while also being considerably faster. Therefore, we conclude that the method can produce extremely reliable results in a very precise amount of time. Several reasons account for the high performance of DPSP:

- It gets diverse information from different aspects of drugs and takes advantage of different types of similarity matrices that yield an inclusive insight into them.
- DPSP uses the summation operator to aggregate the feature vectors of two drugs into one vector for representing the drug–drug pairs in neural network.
- It utilizes a DNN that can extract high-level features from inputs for better prediction and also is easy to implement.

For the additional explanation of the above, different evaluations have been done. First, DPSP uses different features of drugs in order to predict DDIs. Therefore, the number of features is more than the number of samples, and we would like to clarify that this issue has already been addressed using the similarity method. Using this method, the number of features equals the number of samples. To further investigate the effect of dimensionality reduction on our results, we also applied various dimension reduction techniques to all the features obtained in DS1, DS2, and DS3. All of these dimensionality reduction techniques resulted in a performance decrease, which supports the use of the similarity technique.

Second, the choice of aggregation technique is a crucial factor. Similar to what the authors suggested in their previous paper (Masumshah *et al.* 2021), we selected the summation procedure for our research. Specifically, we compared the outcomes of our method to those of the concatenation and dot product methods in Supplementary Tables S40–S42, and discovered that the summation method performed better across all datasets. In addition, we wish to emphasize that the concatenation method depends on the order of the medications, whereas the summation and dot product methods do not. This is an important consideration because the order of the drugs can have an effect on the results, particularly when dealing with large datasets. In contrast, the summation and dot product methods are order-independent, making them more robust and trustworthy in predicting drug interactions. Therefore, we believe that the summation method is a good choice for our method, as it allows us to combine the characteristics of two medications in a simple and intuitive manner, and it has also been demonstrated to be effective in other studies.

Third, DPSP aggregated all the features information and then learned the model using this information. To elucidate, we conducted experiments using various approaches, such as learning a model for each feature and aggregating the results using two distinct approaches: averaging the probabilities for each event and voting on the basis of the most frequent event. However, we discovered that these methods did not perform as well as our primary method, which involves aggregating all feature data and learning the model using these data. On the basis of the data presented in Supplementary Tables S43 and S44, we can conclude that our primary method captures the most pertinent information regarding DDIs and achieves the best possible outcomes.

In all datasets, five events occur most frequently. To further demonstrate DPSP’s ability to predict unknown DDIs, we analyzed DPSP’s predictions for each of these events separately. The results are shown in Supplementary Tables S45–S47. In these tables, the six utilized criteria, including *F*_score, precision, and recall, demonstrate the effectiveness of DPSP in analyzing events from all datasets. For the final evaluation of the presented method, the five FP drug pairs for which the DPSP method gave the highest probability on DS1, DS2, and DS3 were considered in Table 5 and Supplementary Table S48. It should be noted that the version of the Drug Bank used in this study was 5.1.9, but these interactions in Table 5 were added to the most recent version of the Drug Bank, also, interactions in Supplementary Table S48 existed in the TWOSIDES database, which validated the accuracy of these 15 predictions and the performance of the method. As a result, we propose that the method’s other FP with a high probability be considered for further experimental investigations.

**Table 5.** This table displays, for each of the five most frequent events on DS1 and DS2, the new interactions predicted by the DPSP method with the highest probabilities.

	DS1		DS2	
Rank	Drug name 1	Drug name 2	Drug name 1	Drug name 2
1	Amiodarone	Glimepiride	Capsaicin	Fluvoxamine
2	Amiloride	Nabilone	Cyanocobalamin	Aclidinium
3	Agomelatine	Fluvoxamine	Betamethasonephosphate	Cyclosporine
4	Buspirone	Dabrafenib	Tacrolimus	Vitamine
5	Alogliptin	Etacrynicacid	Ranolazine	Crizotinib

According to the findings of this study, using a combination of drug features, a rigorous aggregation schema, and a simple neural network architecture can be an effective method for predicting polypharmacy side effects. For possible future work, we propose utilizing additional characteristics, such as off-label adverse effects and transporter features, as well as various similarity measurements, such as Dice similarity and Cosine similarity.

## Supplementary Material

vbad110_Supplementary_Data
