# Supplementary material for: DPSP: a multimodal deep learning framework for polypharmacy side effects prediction
Source: Bioinform Adv. 2023 Aug 16;3(1):vbad110. doi: 10.1093/bioadv/vbad110 (PMC10493180; doi:10.1093/bioadv/vbad110)
Supplement: vbad110_Supplementary_Data [file vbad110_supplementary_data.pdf]

Table S1. The results of different neural network architectures on DS1.

No. Hidden Layers	No. Neurons	ACC	AUPR	AUROC	F1score	Precision	Recall
1	512	0.9292	0.9743	0.9987	0.9021	0.9021	0.9021
2	512, 256	0.9298	0.9757	0.9988	0.8926	0.8926	0.8926
3	512, 256, 128	**0.9344**	**0.9773**	**0.9990**	**0.9309**	**0.9309**	**0.9309**

Note: The selected model is indicated in bold

Table S2. The results of different neural network architectures on DS2.

No. Hidden Layers	No. Neurons	ACC	AUPR	AUROC	F1score	Precision	Recall
1	512	0.8919	0.9558	0.9991	0.8602	0.8602	0.8602
2	512, 256	0.8986	0.9606	0.9992	0.8532	0.8532	0.8532
3	512, 256, 128	**0.9036**	**0.9633**	**0.9993**	**0.8990**	**0.8990**	**0.8990**

Note: The selected model is indicated in bold

Table S3. The results of different neural network architectures on DS3.

No. Hidden Layers	No. Neurons	ACC	AUPR	AUROC	F1score	Precision	Recall
1	512	0.8329	0.9334	0.9840	0.8354	0.8354	0.8354
2	512, 256	0.8776	0.9414	0.9842	0.8490	0.8490	0.8490
3	512, 256, 128	**0.9100**	**0.9465**	**0.9849**	**0.8558**	**0.8558**	**0.8558**

Note: The selected model is indicated in bold

Table S4. The results of different neural network architectures on DS1.

No. Hidden Layers	No.Neurons	Act.Hid1	Act.Hid2	Act.Hid3	Act.Output	ACC	AUPR	AUROC	F1score	Precision	Recall
3	512, 256, 128	ReLU	ReLU	ReLU	Softmax	0.9330	0.9766	0.9988	0.8528	0.8528	0.8528
3	512, 256, 128	ReLU	ReLU	Sigmoid	Softmax	0.9332	0.9756	0.9990	0.8934	0.8934	0.8934
3	512, 256, 128	ReLU	Sigmoid	Sigmoid	Softmax	**0.9344**	**0.9773**	**0.9990**	**0.9309**	**0.9309**	**0.9309**

Note: The selected model is indicated in bold

Table S5. The results of different neural network architectures on DS2.

No. Hidden Layers	No.Neurons	Act.Hid1	Act.Hid2	Act.Hid3	Act.Output	ACC	AUPR	AUROC	F1score	Precision	Recall
3	512,256,128	ReLU	ReLU	ReLU	Softmax	0.9023	0.9629	0.9993	0.8592	0.8592	0.8592
3	512,256,128	ReLU	ReLU	Sigmoid	Softmax	0.9006	0.9616	0.9992	0.8778	0.8778	0.8778
3	512,256,128	ReLU	Sigmoid	Sigmoid	Softmax	**0.9036**	**0.9633**	**0.9993**	**0.8990**	**0.8990**	**0.8990**

Note: The selected model is indicated in bold

Table S6. The results of different neural network architectures on DS3.

No. Hidden Layers	No.Neurons	Act.Hid1	Act.Hid2	Act.Hid3	Act.Output	ACC	AUPR	AUROC	F1score	Precision	Recall
3	512,256,128	ReLU	ReLU	ReLU	Softmax	0.8977	0.9413	0.9820	0.8253	0.8253	0.8253
3	512,256,128	ReLU	ReLU	Sigmoid	Softmax	0.9015	0.9441	0.9831	0.8490	0.8490	0.8490
3	512,256,128	ReLU	Sigmoid	Sigmoid	Softmax	**0.9100**	**0.9465**	**0.9849**	**0.8558**	**0.8558**	**0.8558**

Note: The selected model is indicated in bold

Table S7. Results of comparison of the DPSP with some of the machine learning methods on DS2.

Method	ACC	AUPR	AUROC	F_ score	Precision	Recall	FP	FN
DPSP	**0.9036**	**0.9633**	**0.9993**	**0.8990**	**0.8990**	**0.8990**	**16,336**	**16,339**
GNN-DDI	0.9020	0.9619	0.9991	0.8900	0.8900	0.8900	17,779	17,779
MSTE	0.8409	0.8959	0.9968	0.8050	0.8050	0.8050	31,529	31,529
MDF-SA-DDI	0.9018	0.9593	0.9991	0.8824	0.8824	0.8824	19,008	19,008
NNPS	0.9006	0.9616	0.9990	0.8777	0.8777	0.8777	19,769	19,769
DDIMDL	0.9019	0.9460	0.9987	0.8915	0.8915	0.8915	17,536	17,536
DNN	0.7908	0.8539	0.9949	0.8045	0.8045	0.8045	31,610	31,610
DeepDDI	0.7211	0.7724	0.9914	0.7178	0.7178	0.7178	45,651	45,651
KNN	0.6932	0.7538	0.9797	0.7408	0.7408	0.7408	41,915	41,915
LR	0.7327	0.7931	0.9955	0.7755	0.7755	0.7755	36,302	36,302
RF	0.7342	0.8028	0.9959	0.7799	0.7799	0.7799	35,590	35,590

Note: Bold numbers show the best performance for each criterion

Table S8. Results of comparison of the DPSP with some of the machine learning methods on DS3.

Method	ACC	AUPR	AUROC	F_ score	Precision	Recall	FP	FN
DPSP	**0.9100**	**0.9465**	**0.9849**	**0.8558**	**0.8558**	**0.8558**	**9,147**	**9,147**
GNN-DDI	0.8989	0.9132	0.9824	0.8483	0.8483	0.8483	9,623	9,623
MSTE	0.8325	0.9004	0.9806	0.8319	0.8319	0.8319	10,664	10,664
MDF-SA-DDI	0.8916	0.9193	0.9799	0.8215	0.8215	0.8215	11,324	11,324
NNPS	0.8413	0.8990	0.9515	0.8324	0.8324	0.8324	10,632	10,632
DDIMDL	0.8771	0.9192	0.9741	0.8239	0.8239	0.8239	11,172	11,172
DNN	0.7708	0.8322	0.9687	0.7919	0.7919	0.7919	13,203	13,203
DeepDDI	0.7522	0.8414	0.9413	0.7258	0.7258	0.7258	17,398	17,398
KNN	0.6999	0.7338	0.9015	0.7079	0.7079	0.7079	18,535	18,535
LR	0.7214	0.7826	0.8998	0.7355	0.7355	0.7355	16,783	16,783
RF	0.7007	0.7645	0.8814	0.7408	0.7408	0.7408	16,446	16,446

Note: Bold numbers show the best performance for each criterion

Table S9. The DPSP method utilized nested cross-validation.

DPSP	ACC	AUPR	AUROC	F_ score	Precision	Recall
DS1	0.9361	0.9785	0.9991	0.9321	0.9321	0.9321
DS2	0.9072	0.9658	0.9994	0.9094	0.9094	0.9094
DS3	0.9120	0.9511	0.9860	0.8573	0.8573	0.8573

Table S10. Determining the impact of removing individual features by computing the true positives (TP) and false positives (FP) for each feature in DS1.

DS1	$TP(F) - TP(F*)$	$TP(F*) - TP(F)$	$TP(F) \cap TP(F*)$	$FP(F) - FP(F*)$	$FP(F*) - FP(F)$	$FP(F) \cap FP(F*)$
F1	1,101	953	33,590	1,132	1,280	1,441
F2	1,005	960	33,686	1,147	1,192	1,426
F3	1,131	1,008	33,560	1,177	1,300	1,396
F4	1,027	977	33,664	1,159	1,209	1,414
F5	1,029	901	33,662	1,081	1,209	1,492

Table S11. This table demonstrates the significance of DS2 data set features by removing one feature and evaluating the results of the DPSP method using the remaining features based on all evaluation criteria.

Excluded feature	ACC	AUPR	AUROC	F_ score	Precision	Recall
Target	0.8922	0.9554	0.9991	0.8694	0.8694	0.8694
Enzyme	0.8998	0.9605	0.9992	0.8688	0.8688	0.8688
Chemical substucture	0.8804	0.9484	0.9990	0.8520	0.8520	0.8520

Table S12. Determining the impact of removing individual features by computing the true positives (TP) and false positives (FP) for each feature in DS2.

DS2	$TP(F') - TP(F'*)$	$TP(F'*) - TP(F')$	$TP(F') \cap TP(F'*)$	$FP(F') - FP(F'*)$	$FP(F'*) - FP(F')$	$FP(F') \cap FP(F'*)$
F'1	6,266	6,088	139,168	7,527	7,705	8,809
F'2	6,987	5,978	138,447	7,485	8,494	8,851
F'3	8,627	5,596	136,807	7,189	10,220	9,147

Table S13. This table demonstrates the significance of DS3 data set features by removing one feature and evaluating the results of the DPSP method using the remaining features based on all evaluation criteria.

Excluded feature	ACC	AUPR	AUROC	F_ score	Precision	Recall
Mono side effect	0.8024	0.9178	0.9750	0.8040	0.8040	0.8040
Target	0.8446	0.9335	0.9813	0.8384	0.8384	0.8384

Table S14. Determining the impact of removing individual features by computing the true positives (TP) and false positives (FP) for each feature in DS3.

DS3	$TP(F'') - TP(F''*)$	$TP(F''*) - TP(F'')$	$TP(F'') \cap TP(F''*)$	$FP(F'') - FP(F''*)$	$FP(F''*) - FP(F'')$	$FP(F'') \cap FP(F''*)$
F"1	3,496	202	50,830	4,740	8,034	4,407
F"2	1,426	315	52,900	5,163	6,274	3,984

Table S15. This table displays the results of the DPSP method utilizing only the pathway feature from DS1.

Feature	ACC	AUPR	AUROC	F_ score	Precision	Recall
Pathway feature	0.8877	0.9321	0.9977	0.8542	0.8542	0.8542

Table S16. This table displays the results of the DPSP method utilizing only the chemical substructure feature from DS2.

Feature	ACC	AUPR	AUROC	F_ score	Precision	Recall
Chemical Substructure	0.8783	0.9466	0.9990	0.8384	0.8384	0.8384

Table S17. This table displays the results of the DPSP method utilizing only the mono side effect feature from DS3.

Feature	ACC	AUPR	AUROC	F_ score	Precision	Recall
Mono side effect	0.7912	0.8988	0.9490	0.7534	0.7534	0.7534

Table S18. The results of performing AutoEncoder (AE) dimensionality reduction technique on DS1.

AE	ACC	AUPR	AUROC	F_ score	Precision	Recall
scenario1	0.7167	0.7923	0.9931	0.6388	0.6388	0.6388
scenario2	0.7928	0.8690	0.9960	0.6851	0.6851	0.6851

Table S19. The results of performing Entropy dimensionality reduction technique on DS1.

Entropy	ACC	AUPR	AUROC	F_ score	Precision	Recall
scenario1	0.6921	0.7702	0.9929	0.6214	0.6214	0.6214
scenario2	0.7365	0.8436	0.9945	0.6432	0.6432	0.6432

Table S20. The results of performing PCA dimensionality reduction technique on DS2.

PCA	ACC	AUPR	AUROC	F_ score	Precision	Recall
scenario1	0.8821	0.9494	0.9991	0.8611	0.8611	0.8611
scenario2	0.7600	0.8410	0.9970	0.7184	0.7184	0.7184

Table S21. The results of performing AutoEncoder (AE) dimensionality reduction technique on DS2.

AE	ACC	AUPR	AUROC	F_ score	Precision	Recall
scenario1	0.7274	0.8022	0.9961	0.6923	0.6923	0.6923
scenario2	0.8013	0.8754	0.9978	0.7414	0.7414	0.7414

Table S22. The results of performing Entropy dimensionality reduction technique on DS2.

Entropy	ACC	AUPR	AUROC	F_ score	Precision	Recall
scenario1	0.7050	0.7798	0.9950	0.6868	0.6868	0.6868
scenario2	0.7999	0.8760	0.9977	0.7401	0.7401	0.7401

Table S23. The results of performing PCA dimensionality reduction technique on DS3.

PCA	ACC	AUPR	AUROC	F_ score	Precision	Recall
scenario1	0.8220	0.8573	0.9798	0.8449	0.8449	0.8449
scenario2	0.7999	0.8456	0.9767	0.8067	0.8067	0.8067

Table S24. The results of performing AutoEncoder (AE) dimensionality reduction technique on DS3.

AE	ACC	AUPR	AUROC	F_ score	Precision	Recall
scenario1	0.7260	0.7669	0.9581	0.7144	0.7144	0.7144
scenario2	0.7484	0.8000	0.9712	0.7369	0.7369	0.7369

Table S25. The results of performing Entropy dimensionality reduction technique on DS3.

Entropy	ACC	AUPR	AUROC	F_ score	Precision	Recall
scenario1	0.6952	0.7489	0.9540	0.6884	0.6884	0.6884
scenario2	0.7328	0.7792	0.9699	0.7035	0.7035	0.7035

Table S26. The results of performing PCA dimensionality reduction technique on all methods in scenario1 on DS1.

PCA	ACC	AUPR	AUROC	F_ score	Precision	Recall
DPSP	0.9264	0.9734	0.9989	0.8919	0.8919	0.8919
GNN-DDI	0.9062	0.9699	0.9979	0.8978	0.8978	0.8978
MSTE	0.8413	0.9251	0.9980	0.8228	0.8228	0.8228
MDF-SA-DDI	0.9035	0.9557	0.9981	0.8703	0.8703	0.8703
NNPS	0.8917	0.9543	0.9978	0.8516	0.8516	0.8516
DDIMDL	0.8666	0.9262	0.9969	0.8020	0.8020	0.8020
DNN	0.8629	0.9023	0.9950	0.7947	0.7947	0.7947
DeepDDI	0.8197	0.8750	0.9956	0.6945	0.6945	0.6945
KNN	0.6982	0.7419	0.9720	0.6934	0.6934	0.6934
LR	0.6920	0.7488	0.9873	0.5044	0.5044	0.5044
RF	0.6956	0.8057	0.9945	0.7013	0.7013	0.7013

Table S27. The results of performing PCA dimensionality reduction technique on all methods in scenario2 on DS1.

PCA	ACC	AUPR	AUROC	F_ score	Precision	Recall
DPSP	0.8942	0.9573	0.9987	0.8279	0.8279	0.8279
GNN-DDI	0.8820	0.9467	0.9969	0.8125	0.8125	0.8125
MSTE	0.8406	0.9236	0.9969	0.8029	0.8029	0.8029
MDF-SA-DDI	0.8923	0.9516	0.9981	0.8121	0.8121	0.8121
NNPS	0.8629	0.9498	0.9968	0.8008	0.8008	0.8008
DDIMDL	0.8449	0.9145	0.9951	0.7990	0.7990	0.7990
DNN	0.8450	0.8907	0.9949	0.7912	0.7912	0.7912
DeepDDI	0.8065	0.8556	0.9934	0.6854	0.6854	0.6854
KNN	0.6787	0.7320	0.9668	0.6960	0.6960	0.6960
LR	0.6718	0.7311	0.9738	0.4912	0.4912	0.4912
RF	0.6704	0.7918	0.9913	0.5168	0.5168	0.5168

Table S28. The results of performing PCA dimensionality reduction technique on all methods in scenario1 on DS2.

PCA	ACC	AUPR	AUROC	F_ score	Precision	Recall
DPSP	0.8821	0.9494	0.9991	0.8611	0.8611	0.8611
GNN-DDI	0.8716	0.9473	0.9989	0.8506	0.8506	0.8506
MSTE	0.8220	0.8778	0.9962	0.7978	0.7978	0.7978
MDF-SA-DDI	0.8974	0.9332	0.9989	0.8527	0.8527	0.8527
NNPS	0.8817	0.9416	0.9990	0.8517	0.8517	0.8517
DDIMDL	0.8800	0.9320	0.9981	0.8428	0.8428	0.8428
DNN	0.7908	0.8539	0.9949	0.7999	0.7999	0.7999
DeepDDI	0.7197	0.7554	0.9901	0.7099	0.7099	0.7099
KNN	0.6824	0.7429	0.9690	0.7311	0.7311	0.7311
LR	0.7160	0.7824	0.9940	0.7563	0.7563	0.7563
RF	0.7129	0.7920	0.9948	0.7697	0.7697	0.7697

Table S29. The results of performing PCA dimensionality reduction technique on all methods in scenario2 on DS2.

PCA	ACC	AUPR	AUROC	F_ score	Precision	Recall
DPSP	0.7600	0.8410	0.9970	0.7184	0.7184	0.7184
GNN-DDI	0.7534	0.8315	0.9970	0.7098	0.7098	0.7098
MSTE	0.7172	0.7750	0.9934	0.6984	0.6984	0.6984
MDF-SA-DDI	0.7598	0.8312	0.9967	0.7020	0.7020	0.7020
NNPS	0.7517	0.8365	0.9956	0.7123	0.7123	0.7123
DDIMDL	0.7566	0.8320	0.9951	0.6990	0.6990	0.6990
DNN	0.7114	0.7359	0.9929	0.6850	0.6850	0.6850
DeepDDI	0.6834	0.7222	0.9824	0.6625	0.6625	0.6625
KNN	0.6547	0.7119	0.9589	0.6920	0.6920	0.6920
LR	0.6971	0.7324	0.9913	0.7018	0.7018	0.7018
RF	0.6949	0.7350	0.9918	0.7130	0.7130	0.7130

Table S30. The results of performing PCA dimensionality reduction technique on all methods in scenario1 on DS3.

PCA	ACC	AUPR	AUROC	F_ score	Precision	Recall
DPSP	0.8220	0.8573	0.9798	0.8449	0.8449	0.8449
GNN-DDI	0.8003	0.8223	0.9623	0.8320	0.8320	0.8320
MSTE	0.7460	0.8139	0.9600	0.8299	0.8299	0.8299
MDF-SA-DDI	0.8016	0.8239	0.9615	0.8162	0.8162	0.8162
NNPS	0.7571	0.8090	0.9420	0.8150	0.8150	0.8150
DDIMDL	0.6814	0.8222	0.9616	0.8100	0.8100	0.8100
DNN	0.6822	0.7492	0.9558	0.7734	0.7734	0.7734
DeepDDI	0.6613	0.7514	0.9227	0.7215	0.7215	0.7215
KNN	0.6099	0.6466	0.9000	0.6864	0.6864	0.6864
LR	0.6307	0.6945	0.8814	0.6540	0.6540	0.6540
RF	0.6182	0.6726	0.8794	0.6729	0.6729	0.6729

Table S31. The results of performing PCA dimensionality reduction technique on all methods in scenario2 on DS3.

PCA	ACC	AUPR	AUROC	F_ score	Precision	Recall
DPSP	0.7999	0.8456	0.9767	0.8067	0.8067	0.8067
GNN-DDI	0.7622	0.8335	0.9554	0.7971	0.7971	0.7971
MSTE	0.7219	0.8027	0.9416	0.7558	0.7558	0.7558
MDF-SA-DDI	0.7925	0.8174	0.9542	0.7711	0.7711	0.7711
NNPS	0.7369	0.7989	0.9387	0.7346	0.7346	0.7346
DDIMDL	0.6558	0.8147	0.9528	0.7474	0.7474	0.7474
DNN	0.6707	0.7355	0.9430	0.7580	0.7580	0.7580
DeepDDI	0.6520	0.7400	0.9160	0.7437	0.7437	0.7437
KNN	0.6025	0.6557	0.8878	0.6529	0.6529	0.6529
LR	0.6276	0.6832	0.8904	0.6464	0.6464	0.6464
RF	0.6171	0.6599	0.8852	0.6638	0.6638	0.6638

Table S32. Data availability for each feature type in DS1.

Feature	No. Zero elements before similarity	No. Zero elements after similarity	No. Nonzero elements before similarity	No. Nonzero elements after similarity
Mono side effect	5,557,163	2,658	157,689	324,526
Target	661,618	311,184	3,046	16,000
Enzyme	113,411	166,570	2,133	160,614
Pathway	544,627	311,370	2,777	15,814
Chemical substructure	433,582	602	70,350	326,582

Table S33. Data availability for each feature type in DS2.

Feature	No. Zero elements before similarity	No. Zero elements after similarity	No. Nonzero elements before similarity	No. Nonzero elements after similarity
Target	2,071,350	1,522,590	5,608	59,974
Enzyme	393,177	793,384	4,351	789,180
Chemical substructure	2,507,884	18,502	58,436	1,564,062

Table S34. Data availability for each feature type in DS3.

Feature	No. Zero elements before similarity	No. Zero elements after similarity	No. Nonzero elements before similarity	No. Nonzero elements after similarity
Mono side effect	6,393,703	11,110	174,977	404,915
Target	5,743,740	263,208	18,690	152,817

Table S35. Effect of different feature combinations in DS1 on performance of model We used symbols (M for mono side effects, T for targets, E for enzymes, P for pathways, and S for smiles features) to represent the different types of features (part1).

Rank	Feature set	ACC	AUPR	AUROC	F_ score	Precision	Recall
1	M+T+E+P+S	**0.9344**	**0.9773**	**0.9990**	**0.9309**	**0.9309**	**0.9309**
2	M+T+E+S+P	0.9300	0.9750	0.9990	0.8845	0.8845	0.8845
3	M+T+P+E+S	0.9298	0.9754	0.9990	0.9036	0.9036	0.9036
4	M+T+P+S+E	0.9303	0.9762	0.9990	0.8898	0.8898	0.8898
5	M+T+S+E+P	0.9329	0.9759	0.9990	0.8866	0.8866	0.8866
6	M+T+S+P+E	0.9311	0.9757	0.9990	0.8763	0.8763	0.8763
7	M+E+T+P+S	0.9313	0.9751	0.9989	0.9029	0.9029	0.9029
8	M+E+T+S+P	0.9311	0.9753	0.9989	0.8888	0.8888	0.8888
9	M+E+P+T+S	0.9299	0.9752	0.9990	0.8937	0.8937	0.8937
10	M+E+P+S+T	0.9316	0.9758	0.9990	0.8992	0.8992	0.8992
11	M+E+S+T+P	0.9308	0.9753	0.9989	0.8925	0.8925	0.8925
12	M+E+S+P+T	0.9298	0.9748	0.9989	0.8945	0.8945	0.8945
13	M+P+T+E+S	0.9306	0.9753	0.9990	0.8833	0.8833	0.8833
14	M+P+T+S+E	0.9297	0.9744	0.9989	0.8822	0.8822	0.8822
15	M+P+E+T+S	0.9311	0.9752	0.9989	0.9085	0.9085	0.9085
16	M+P+E+S+T	0.9216	0.9623	0.9970	0.8873	0.8873	0.8873
17	M+P+S+T+E	0.9320	0.9754	0.9989	0.9100	0.9100	0.9100
18	M+P+S+E+T	0.9309	0.9713	0.9988	0.8999	0.8999	0.8999
19	M+S+T+E+P	0.9323	0.9764	0.9990	0.9000	0.9000	0.9000
20	M+S+T+P+E	0.9299	0.9699	0.9989	0.8988	0.8988	0.8988
21	M+S+E+T+P	0.9300	0.9701	0.9990	0.9115	0.9115	0.9115
22	M+S+E+P+T	0.9190	0.9514	0.9989	0.8716	0.8716	0.8716
23	M+S+P+T+E	0.9245	0.9683	0.9989	0.8820	0.8820	0.8820
24	M+S+P+E+T	0.9256	0.9698	0.9989	0.8834	0.8834	0.8834
25	T+M+E+P+S	0.9312	0.9714	0.9990	0.9125	0.9125	0.9125
26	T+M+E+S+P	0.9269	0.9580	0.9989	0.9064	0.9064	0.9064
27	T+M+P+E+S	0.9315	0.9699	0.9990	0.9121	0.9121	0.9121
28	T+M+P+S+E	0.9318	0.9705	0.9990	0.9113	0.9113	0.9113
29	T+M+S+E+P	0.9288	0.9636	0.9989	0.9028	0.9028	0.9028
30	T+M+S+P+E	0.9326	0.9729	0.9990	0.9142	0.9142	0.9142
31	T+E+M+P+S	0.9157	0.9536	0.9988	0.8917	0.8917	0.8917
32	T+E+M+S+P	0.9150	0.9514	0.9988	0.8920	0.8920	0.8920
33	T+E+P+M+S	0.9260	0.9639	0.9989	0.8965	0.8965	0.8965
34	T+E+P+S+M	0.9311	0.9710	0.9990	0.9111	0.9111	0.9111
35	T+E+S+M+P	0.9336	0.9740	0.9990	0.9240	0.9240	0.9240
36	T+E+S+P+M	0.9317	0.9709	0.9990	0.9156	0.9156	0.9156
37	T+P+M+E+S	0.9018	0.9514	0.9987	0.8717	0.8717	0.8717
38	T+P+M+S+E	0.9126	0.9616	0.9988	0.8823	0.8823	0.8823
39	T+P+E+M+S	0.9049	0.9587	0.9987	0.8806	0.8806	0.8806
40	T+P+E+S+M	0.9314	0.9703	0.9990	0.9089	0.9089	0.9089
41	T+P+S+M+E	0.9263	0.9651	0.9989	0.8961	0.8961	0.8961
42	T+P+S+E+M	0.9255	0.9650	0.9989	0.8957	0.8957	0.8957
43	T+S+M+E+P	0.9278	0.9669	0.9989	0.8963	0.8963	0.8963
44	T+S+M+P+E	0.9268	0.9677	0.9989	0.8960	0.8960	0.8960
45	T+S+E+M+P	0.9299	0.9701	0.9989	0.8974	0.8974	0.8974
46	T+S+E+P+M	0.9310	0.9722	0.9990	0.9121	0.9121	0.9121
47	T+S+P+M+E	0.9311	0.9757	0.9990	0.9118	0.9118	0.9118
48	T+S+P+E+M	0.9321	0.9761	0.9990	0.9142	0.9142	0.9142
49	E+M+T+P+S	0.9164	0.9684	0.9987	0.8749	0.8749	0.8749
50	E+M+T+S+P	0.9095	0.9578	0.9986	0.8635	0.8635	0.8635
51	E+M+P+T+S	0.9187	0.9634	0.9987	0.8670	0.8670	0.8670
52	E+M+P+S+T	0.9198	0.9623	0.9988	0.8684	0.8684	0.8684
53	E+M+S+T+P	0.9090	0.9512	0.9987	0.8598	0.8598	0.8598
54	E+M+S+P+T	0.9142	0.9599	0.9988	0.8690	0.8690	0.8690
55	E+T+M+P+S	0.9136	0.9598	0.9988	0.8688	0.8688	0.8688
56	E+T+M+S+P	0.9028	0.9646	0.9987	0.8593	0.8593	0.8593
57	E+T+P+M+S	0.9036	0.9554	0.9987	0.8600	0.8600	0.8600
58	E+T+P+S+M	0.9113	0.9599	0.9988	0.8613	0.8613	0.8613
59	E+T+S+M+P	0.9046	0.9620	0.9987	0.8602	0.8602	0.8602
60	E+T+S+P+M	0.9052	0.9615	0.9987	0.8607	0.8607	0.8607

Note: Bold numbers show the best performance for each criterion

Table S36. Effect of different feature combinations in DS1 on performance of model We used symbols (M for mono side effects, T for targets, E for enzymes, P for pathways, and S for smiles features) to represent the different types of features (part2).

Rank	Feature set	ACC	AUPR	AUROC	F_ score	Precision	Recall
61	E+P+M+T+S	0.9074	0.9652	0.9987	0.8817	0.8817	0.8817
62	E+P+M+S+T	0.9147	0.9680	0.9988	0.9021	0.9021	0.9021
63	E+P+T+M+S	0.9216	0.9700	0.9988	0.8989	0.8989	0.8989
64	E+P+T+S+M	0.9220	0.9706	0.9988	0.8991	0.8991	0.8991
65	E+P+S+M+T	0.9118	0.9699	0.9987	0.8975	0.8975	0.8975
66	E+P+S+T+M	0.9250	0.9714	0.9989	0.9010	0.9010	0.9010
67	E+S+M+T+P	0.9249	0.9712	0.9989	0.9009	0.9009	0.9009
68	E+S+M+P+T	0.9122	0.9699	0.9988	0.8999	0.8999	0.8999
69	E+S+T+M+P	0.9113	0.9633	0.9988	0.8840	0.8840	0.8840
70	E+S+T+P+M	0.9116	0.9635	0.9988	0.8842	0.8842	0.8842
71	E+S+P+M+T	0.9015	0.9601	0.9987	0.8778	0.8778	0.8778
72	E+S+P+T+M	0.9009	0.9599	0.9987	0.8775	0.8775	0.8775
73	P+M+T+E+S	0.8999	0.9470	0.9898	0.8420	0.8420	0.8420
74	P+M+T+S+E	0.8999	0.9472	0.9898	0.8432	0.8432	0.8432
75	P+M+E+T+S	0.8988	0.9423	0.9897	0.8420	0.8420	0.8420
76	P+M+E+S+T	0.8979	0.9420	0.9897	0.8419	0.8419	0.8419
77	P+M+S+E+T	0.8980	0.9422	0.9897	0.8434	0.8434	0.8434
78	P+M+S+T+E	0.8980	0.9421	0.9897	0.8426	0.8426	0.8426
79	P+T+M+E+S	0.8870	0.9312	0.9716	0.8324	0.8324	0.8324
80	P+T+M+S+E	0.8866	0.9309	0.9714	0.8322	0.8322	0.8322
81	P+T+E+M+S	0.8856	0.9300	0.9709	0.0.8319	0.8319	0.8319
82	P+T+E+S+M	0.8860	0.9302	0.9711	0.8323	0.8323	0.8323
83	P+T+S+M+E	0.8861	0.9303	0.9714	0.8324	0.8324	0.8324
84	P+T+S+E+M	0.8860	0.9301	0.9714	0.8322	0.8322	0.8322
85	P+E+M+T+S	0.8863	0.9305	0.9713	0.8324	0.8324	0.8324
86	P+E+M+S+T	0.8864	0.9305	0.9713	0.8325	0.8325	0.8325
87	P+E+T+M+S	0.8866	0.9302	0.9709	0.8303	0.8303	0.8303
88	P+E+T+S+M	0.8859	0.9303	0.9709	0.8305	0.8305	0.8305
89	P+E+S+M+T	0.8859	0.9301	0.9709	0.8301	0.8301	0.8301
90	P+E+S+T+M	0.8849	0.9300	0.9713	0.8312	0.8312	0.8312
91	P+S+M+T+E	0.8799	0.9289	0.9709	0.8300	0.8300	0.8300
92	P+S+M+E+T	0.8800	0.9292	0.9710	0.8305	0.8305	0.8305
93	P+S+T+M+E	0.8801	0.9292	0.9710	0.8306	0.8306	0.8306
94	P+S+T+E+M	0.8798	0.9290	0.9709	0.8299	0.8299	0.8299
95	P+S+E+M+T	0.8799	0.9291	0.9709	0.8299	0.8299	0.8299
96	P+S+E+T+M	0.8798	0.9290	0.9709	0.8298	0.8298	0.8298
97	S+M+T+E+P	0.8800	0.9292	0.9710	0.8300	0.8300	0.8300
98	S+M+T+P+E	0.8801	0.9350	0.9700	0.8121	0.8121	0.8121
99	S+M+E+T+P	0.8693	0.9300	0.9604	0.8060	0.8060	0.8060
100	S+M+E+P+T	0.8695	0.9300	0.9605	0.8062	0.8062	0.8062
101	S+M+P+T+E	0.8699	0.9302	0.9610	0.8071	0.8071	0.8071
102	S+M+P+E+T	0.8701	0.9309	0.9611	0.8079	0.8079	0.8079
103	S+T+M+E+P	0.8699	0.9305	0.9615	0.8075	0.8075	0.8075
104	S+T+M+P+E	0.8720	0.9324	0.9717	0.8152	0.8152	0.8152
105	S+T+E+M+P	0.8721	0.9325	0.9717	0.8155	0.8155	0.8155
106	S+T+E+P+M	0.8716	0.9319	0.9716	0.8140	0.8140	0.8140
107	S+T+P+M+E	0.8710	0.9317	0.9716	0.8139	0.8139	0.8139
108	S+T+P+E+M	0.8690	0.9280	0.9601	0.8059	0.8059	0.8059
109	S+E+M+T+P	0.8690	0.9299	0.9600	0.8060	0.8060	0.8060
110	S+E+M+P+T	0.8760	0.9407	0.9670	0.8190	0.8190	0.8190
111	S+E+T+M+P	0.8763	0.9409	0.9675	0.8192	0.8192	0.8192
112	S+E+T+P+M	0.8702	0.9310	0.9605	0.8051	0.8051	0.8051
113	S+E+P+M+T	0.8730	0.9329	0.9646	0.8105	0.8105	0.8105
114	S+E+P+T+M	0.8721	0.9322	0.9624	0.8100	0.8100	0.8100
115	S+P+M+T+E	0.8759	0.9402	0.9672	0.8190	0.8190	0.8190
116	S+P+M+E+T	0.8709	0.9314	0.9615	0.8063	0.8063	0.8063
117	S+P+T+M+E	0.8799	0.9390	0.9662	0.8130	0.8130	0.8130
118	S+P+T+E+M	0.8715	0.9321	0.9619	0.8080	0.8080	0.8080
119	S+P+E+M+T	0.8734	0.9330	0.9650	0.8125	0.8125	0.8125
120	S+P+E+T+M	0.8716	0.9322	0.9620	0.8085	0.8085	0.8085

Table S37. Effect of different feature combinations in DS2 on performance of model We used symbols (T for targets, E for enzymes, and S for smiles features) to represent the different types of features.

Rank	Feature set	ACC	AUPR	AUROC	F_ score	Precision	Recall
1	T+E+S	**0.9036**	**0.9633**	**0.9993**	**0.8990**	**0.8990**	**0.8990**
2	T+S+E	0.8669	0.9385	0.9988	0.8454	0.8454	0.8454
3	E+T+S	0.8679	0.9389	0.9988	0.8373	0.8373	0.8373
4	E+S+T	0.9003	0.9609	0.9992	0.8741	0.8741	0.8741
5	S+E+T	0.8712	0.9401	0.9988	0.8427	0.8427	0.8427
6	S+T+E	0.9002	0.9612	0.9992	0.8771	0.8771	0.8771

Note: Bold numbers show the best performance for each criterion

Table S38. Effect of different feature combinations in DS3 on performance of model We used symbols (M for mono side effects, T for targets features) to represent the different types of features.

Rank	Feature set	ACC	AUPR	AUROC	F_ score	Precision	Recall
1	M+T	**0.9100**	**0.9465**	**0.9849**	**0.8558**	**0.8558**	**0.8558**
2	T+M	0.8614	0.9123	0.9670	0.8116	0.8116	0.8116

Note: Bold numbers show the best performance for each criterion

Table S39. Comparison results between the execution time of the DPSP method and other machine learning methods on all datasets.

Dataset	DPSP	GNN-DDI	MSTE	MDF-SA-DDI	NNPS	DDIMDL	DNN	DeepDDI	KNN	LR	RF
DS1	**20 min**	90 min	1440 min	120 min	45 min	70 min	40 min	60 min	31 min	40 min	50 min
DS2	**120 min**	240 min	4320 min	276 min	141 min	289 min	110 min	140 min	153 min	205 min	260 min
DS3	**25 min**	110 min	2160	150 min	120 min	75 min	50 min	70 min	40 min	50 min	60 min

Table S40. The process of selecting the method for aggregating input features in the neural network in DS1.

DS1	ACC	AUPR	AUROC	F_ score	Precision	Recall
Summation	**0.9344**	**0.9773**	**0.9990**	**0.9309**	**0.9309**	**0.9309**
Concatenation	0.8915	0.9479	0.9976	0.8453	0.8453	0.8453
Dot product	0.5384	0.5590	0.9796	0.4834	0.4834	0.4834

Note: Bold numbers show the best performance for each criterion

Table S41. The process of selecting the method for aggregating input features in the neural network in DS2.

DS2	ACC	AUPR	AUROC	F_ score	Precision	Recall
Summation	**0.9036**	**0.9633**	**0.9993**	**0.8990**	**0.8990**	**0.8990**
Concatenation	0.8690	0.9628	0.9992	0.8837	0.8837	0.8837
Dot product	0.5150	0.5162	0.9809	0.4693	0.4693	0.4693

Note: Bold numbers show the best performance for each criterion

Table S42. The process of selecting the method for aggregating input features in the neural network in DS3.

DS3	ACC	AUPR	AUROC	F_ score	Precision	Recall
Summation	**0.9100**	**0.9465**	**0.9849**	**0.8558**	**0.8558**	**0.8558**
Concatenation	0.8514	0.9420	0.9839	0.8401	0.8401	0.8401
Dot product	0.5016	0.5143	0.9790	0.4516	0.4516	0.4516

Note: Bold numbers show the best performance for each criterion

Table S43. The process of feature learning based on averaging the outputs on DS1, DS2, and DS3.

Average	ACC	AUPR	AUROC	F_ score	Precision	Recall
DS1	0.9247	0.9667	0.9989	0.8926	0.8926	0.8926
DS2	0.8856	0.9322	0.9986	0.8788	0.8788	0.8788
DS3	0.8714	0.9209	0.9980	0.8817	0.8817	0.8817

Table S44. The process of feature learning based on voting the outputs on DS1, DS2, and DS3.

Vote	ACC	AUPR	AUROC	F_ score	Precision	Recall
DS1	0.9168	0.7818	0.9928	0.8829	0.8829	0.8829
DS2	0.8658	0.7520	0.9916	0.8411	0.8411	0.8411
DS3	0.8525	0.7362	0.9899	0.8297	0.8297	0.8297

Table S45. This table displays the performance of the DPSP method on the five most common drug combinations in DS1 according to all evaluation criteria.

Rank	Event Name	ACC	AUPR	AUROC	F_ score	Precision	Recall
1	Decreasing the metabolism	0.9680	0.9466	0.9617	0.9398	0.9398	0.9398
2	Increasing the risk or severity of adverse effects	0.9792	0.9644	0.9729	0.9586	0.9586	0.9586
3	Increasing the serum concentration	0.9718	0.9140	0.9422	0.9131	0.9131	0.9131
4	Decreasing the serum concentration	0.9894	0.9198	0.9475	0.9338	0.9338	0.9338
5	Decreasin the therapeutic efficacy	0.9957	0.9407	0.9577	0.9616	0.9616	0.9616

Table S46. This table displays the performance of the DPSP method on the five most common drug combinations in DS2 according to all evaluation criteria.

Rank	Event Name	ACC	AUPR	AUROC	F_ score	Precision	Recall
1	Decreasing the metabolism	0.9449	0.9253	0.9366	0.9144	0.9144	0.9144
2	Decreasing the excretion rate which could result in a higher serum level	0.9825	0.9287	0.9551	0.9287	0.9287	0.9287
3	Increasing the risk or severity of adverse effects	0.9826	0.9225	0.9549	0.9197	0.9197	0.9197
4	Increasing the metabolism	0.9807	0.8880	0.9390	0.8890	0.8890	0.8890
5	Increasing the serum concentration	0.9789	0.8954	0.9786	0.9120	0.9120	0.9120

Table S47. This table displays the performance of the DPSP method on the five most common drug combinations in DS3 according to all evaluation criteria.

Rank	Event Name	ACC	AUPR	AUROC	F_ score	Precision	Recall
1	Arterial pressure NOS decreased	0.9113	0.9353	0.9821	0.8600	0.8600	0.8600
2	Anaemia	0.9121	0.9357	0.9845	0.8832	0.8832	0.8832
3	Difficulty breathing	0.9134	0.9416	0.9848	0.8907	0.8907	0.8907
4	Nausea	0.9117	0.9356	0.9834	0.8724	0.8724	0.8724
5	Neumonia	0.9180	0.9403	0.9849	0.9011	0.9011	0.9011

Table S48. This table displays, for each of the five most frequent events on DS3, the new interactions predicted by the DPSP method with the highest probabilities.

Rank	Drug Name1	Drug Name2
1	Ampicillin	Fentanyl
2	Orphenadrine	Tamoxifen
3	Rofecoxib	Triazolam
4	Propranolol	Olmesartan medoxomil
5	Temazepam	Trimipramine

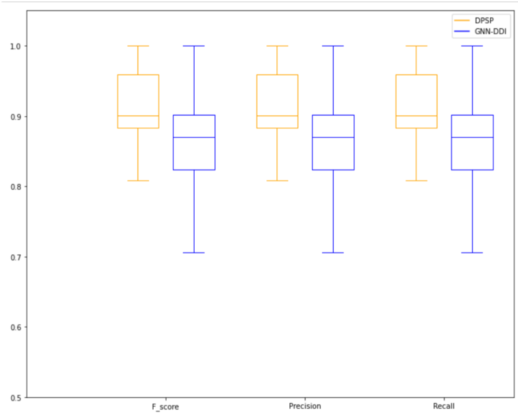

Fig. S1. Boxplots of F_ score, Precision ,and Recall values of 65 events on DS1 for DPSP and GNN-DDI methods.

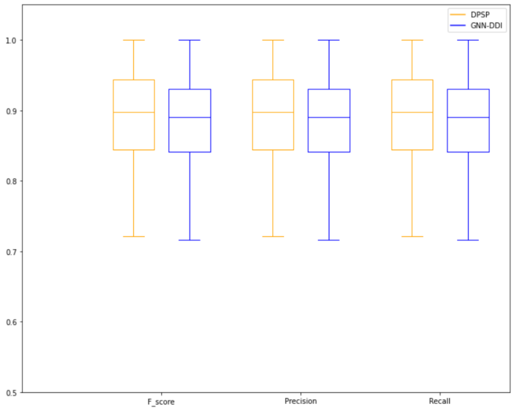

Fig. S2. Boxplots of F_ score, Precision ,and Recall values of 100 events on DS2 for DPSP and GNN-DDI methods.

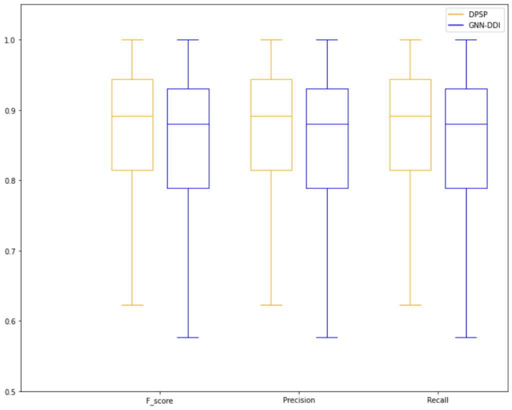

Fig. S3. Boxplots of F_ score, Precision ,and Recall values of 409 events on DS3 for DPSP and GNN-DDI methods.

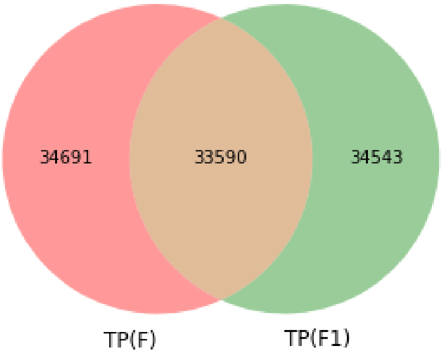

Fig. S4. Venn diagram of comaring the impact of results of the method using all features (F) with excluding mono side effect feature (F1) in DS1 by computing true positive (TP).

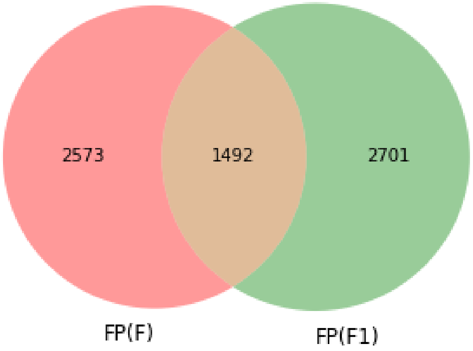

Fig. S5. Venn diagram of comaring the impact of results of the method using all features (F) with excluding mono side effect feature (F1) in DS1 by computing false positive (FP).

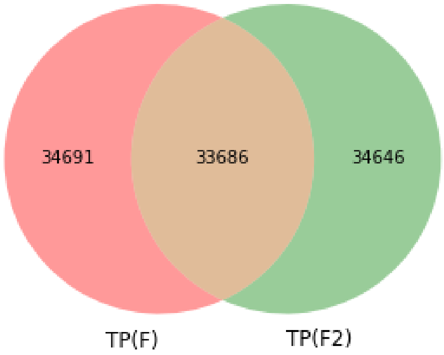

Fig. S6. Venn diagram of comaring the impact of results of the method using all features (F) with excluding target feature (F2) in DS1 by computing true positive (TP).

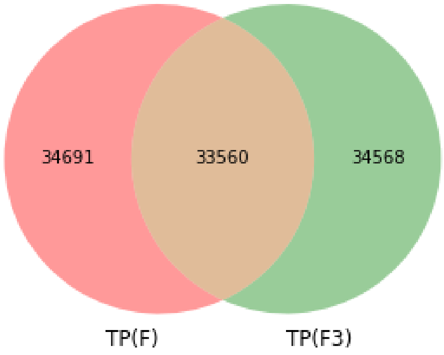

Fig. S8. Venn diagram of comaring the impact of results of the method using all features (F) with excluding enzyme feature (F3) in DS1 by computing true positive (TP).

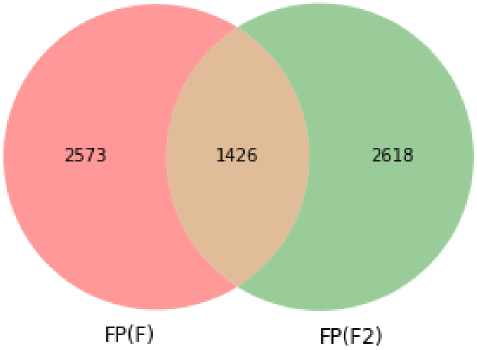

Fig. S7. Venn diagram of comaring the impact of results of the method using all features (F) with excluding target feature (F2) in DS1 by computing false positive (FP).

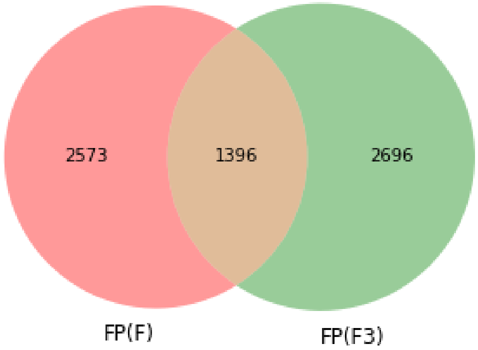

Fig. S9. Venn diagram of comaring the impact of results of the method using all features (F) with excluding enzyme feature (F3) in DS1 by computing false positive (FP).

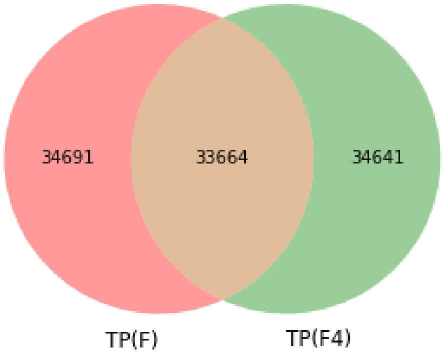

Fig. S10. Venn diagram of comaring the impact of results of the method using all features (F) with excluding chemical substructure feature (F4) in DS1 by computing true positive (TP).

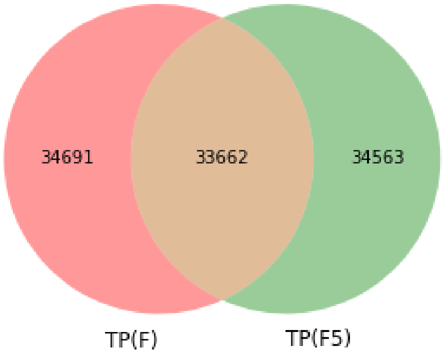

Fig. S12. Venn diagram of comaring the impact of results of the method using all features (F) with excluding pathway feature (F5) in DS1 by computing true positive (TP).

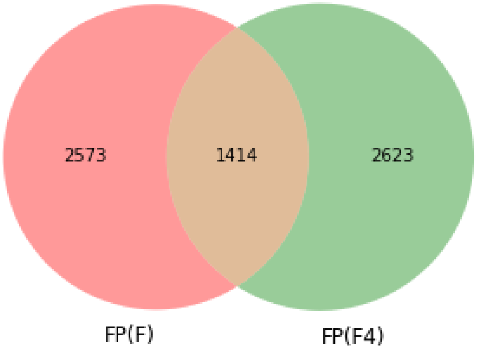

Fig. S11. Venn diagram of comaring the impact of results of the method using all features (F) with excluding chemical substructure feature (F4) in DS1 by computing false positive (FP).

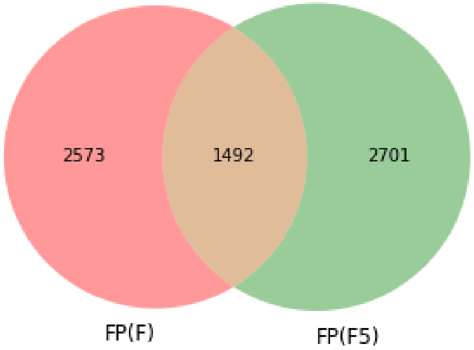

Fig. S13. Venn diagram of comaring the impact of results of the method using all features (F) with excluding pathway feature (F5) in DS1 by computing false positive (FP).

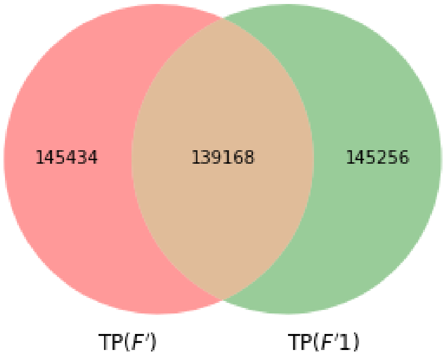

Fig. S14. Venn diagram of comaring the impact of results of the method using all features (F') with excluding target feature (F'1) in DS2 by computing true positive (TP).

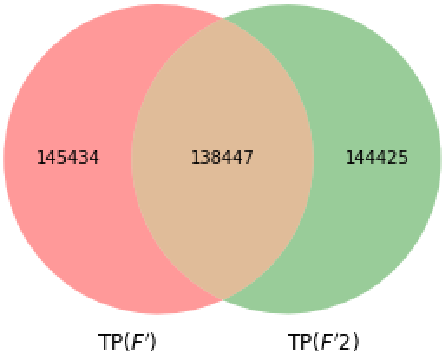

Fig. S16. Venn diagram of comaring the impact of results of the method using all features (F') with excluding enzyme feature (F'2) in DS2 by computing true positive (TP).

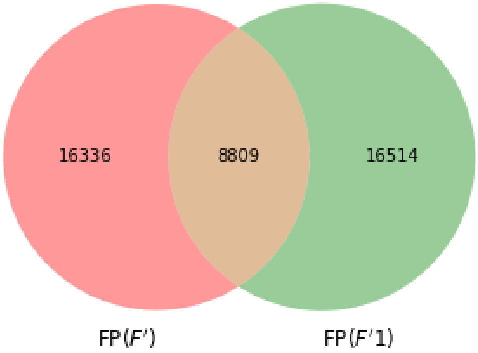

Fig. S15. Venn diagram of comaring the impact of results of the method using all features (F') with excluding target feature (F'1) in DS2 by computing false positive (FP).

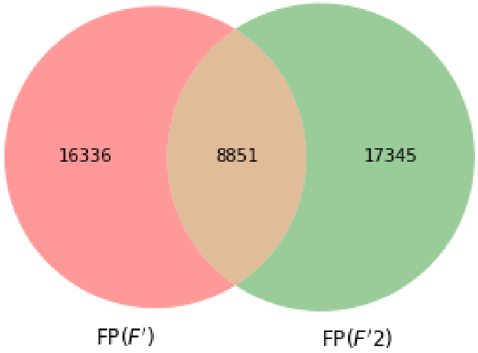

Fig. S17. Venn diagram of comaring the impact of results of the method using all features (F') with excluding enzyme feature (F'2) in DS2 by computing false positive (FP).

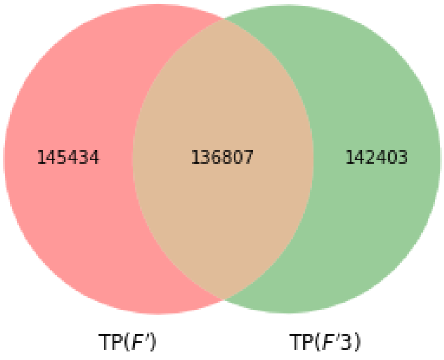

Fig. S18. Venn diagram of comaring the impact of results of the method using all features (F') with excluding chemical substructure feature (F'3) in DS2 by computing true positive (TP).

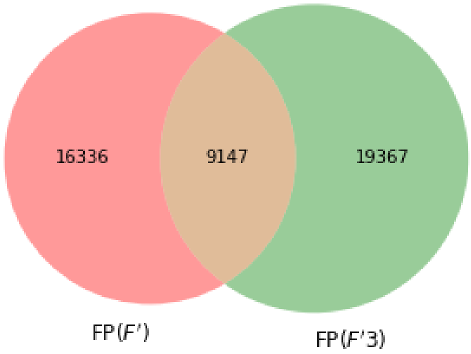

Fig. S19. Venn diagram of comaring the impact of results of the method using all features (F') with excluding chemical substructure feature (F'3) in DS2 by computing false positive (FP).

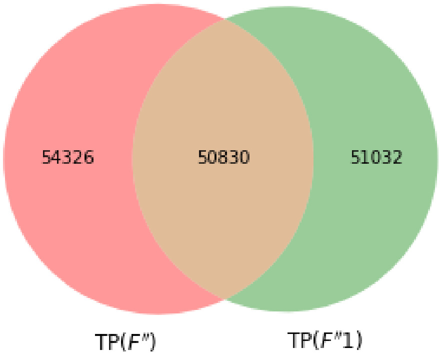

Fig. S20. Venn diagram of comaring the impact of results of the method using all features (F") with excluding mono side effect feature (F"1) in DS3 by computing true positive (TP).

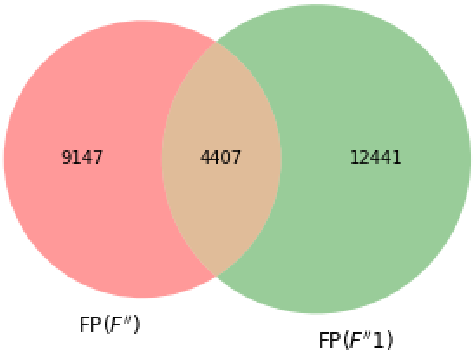

Fig. S21. Venn diagram of comaring the impact of results of the method using all features (F") with excluding mono side effect feature (F"1) in DS3 by computing false positive (FP).

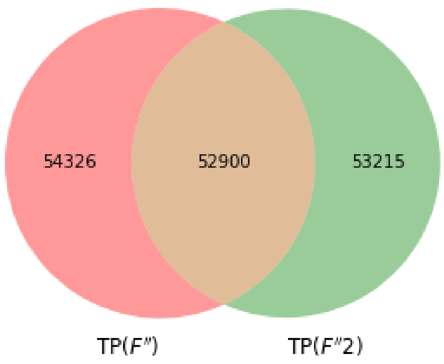

Fig. S22. Venn diagram of comaring the impact of results of the method using all features (F”) with excluding target feature (F”2) in DS3 by computing true positive (TP).

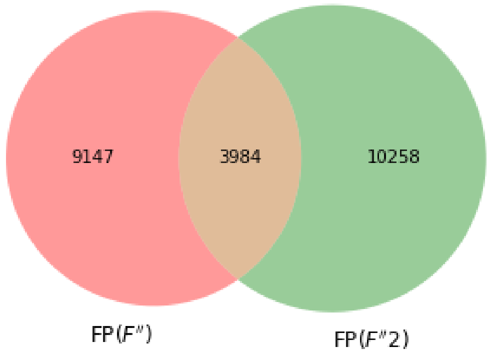

Fig. S23. Venn diagram of comaring the impact of results of the method using all features (F”) with excluding target feature (F”2) in DS3 by computing false positive (FP).
